# Land‐use change shifts and magnifies seasonal variations of the decomposer system in lowland tropical landscapes

**DOI:** 10.1002/ece3.9020

**Published:** 2022-06-17

**Authors:** Valentyna Krashevska, Christian Stiegler, Tania June, Rahayu Widyastuti, Alexander Knohl, Stefan Scheu, Anton Potapov

**Affiliations:** ^1^ J.F. Blumenbach Institute of Zoology and Anthropology University of Göttingen Göttingen Germany; ^2^ Bioclimatology University of Göttingen Göttingen Germany; ^3^ Department of Geophysics and Meteorology Bogor Agricultural University (IPB) Bogor Indonesia; ^4^ Department of Soil Sciences and Land Resources Bogor Agricultural University (IPB) Bogor Indonesia; ^5^ Centre of Biodiversity and Sustainable Land Use University of Göttingen Göttingen Germany

**Keywords:** animals, climate, decomposers, deforestation, land‐use change, microorganisms, plantations, rainforest, seasonality

## Abstract

Deforestation and agricultural expansion in the tropics affect local and regional climatic conditions, leading to synergistic negative impacts on land ecosystems. Climatic changes manifest in increased inter‐ and intraseasonal variations and frequency of extreme climatic events (i.e., droughts and floods), which have evident consequences for aboveground biodiversity. However, until today, there have been no studies on how land use affects seasonal variations below ground in tropical ecosystems, which may be more buffered against climatic variation. Here, we analyzed seasonal variations in soil parameters, basal respiration, microbial communities, and abundances of soil invertebrates along with microclimatic conditions in rainforest and monocultures of oil palm and rubber in Sumatra, Indonesia. About 75% (20 out of 26) of the measured litter and soil, microbial, and animal parameters varied with season, with seasonal changes in 50% of the parameters depending on land use. Land use affected seasonal variations in microbial indicators associated with carbon availability and cycling rate. The magnitude of seasonal variations in microbial parameters in the soil of monocultures was almost 40% higher than in the soil of rainforest. Measured parameters were associated with short‐term climatic conditions (3‐day period air humidity) in plantations, but not in rainforest, confirming a reduced soil buffering ability in plantations. Overall, our findings suggest that land use temporally shifts and increases the magnitude of seasonal variations of the belowground ecosystem compartment, with microbial communities responding most strongly. The increased seasonal variations in soil biota in plantations likely translate into more pronounced fluctuations in essential ecosystem functions such as nutrient cycling and carbon sequestration, and these ramifications ultimately may compromise the stability of tropical ecosystems in the long term. As the observed seasonal dynamics is likely to increase with both local and global climate change, these shifts need closer attention for the long‐term sustainable management of plantation systems in the tropics.

## Graphical abstract

Land use shifted seasonal changes in microbial, animal, and bulk soil parameters. The magnitude of soil microbial variation increased by 40% in monocultures. The soil system in monocultures is affected by short‐term microclimatic changes. Increased seasonal variation in soil functioning is an aspect of global change.

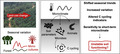

## 1 INTRODUCTION

Land use and climatic variations are arguably the two most critical drivers of ecosystem changes worldwide (IPBES, 2019). Deforestation and agricultural expansion across equatorial lowland regions are driven by the high global demand for low‐cost oils, such as palm oil, and are expected to further increase in the near future (Miettinen et al., 2012; Pirker et al., 2016; Sumarga & Hein, 2016). Compared to rainforest and other vegetation‐ and species‐rich ecosystems, monoculture plantations are less resilient to both seasonal variations in climate and climate extremes (Hutchison et al., 2018; Kunert & Cárdenas, 2015). In monoculture plantations, decreased or increased precipitation rates and prolonged dry seasons may have consequent effects on various ecosystem components. For example, oil palm monoculture plantations are commonly covered by vegetation with mainly shallow roots (Zuraidah et al., 2010), which limits their access to deepwater sources resulting in low drought tolerance. Further, global climate change is expected to increase the variability of the El Niño‐Southern Oscillation (ENSO), the most dominant climate variation on Earth, resulting in prolonged drought periods and increased precipitation variability in the eastern Pacific region (Cai et al., 2018; Keupp et al., 2017). These predicted changes may severely impact rainforest and monoculture plantations, for example, increasing the area unsuitable for growing oil palm (Paterson et al., 2017), and reducing rainforest ecosystem services, productivity, resilience, and biodiversity (Paterson et al., 2017). While the effects of seasonal and experimental water supply and fluctuation were intensively studied in tropical forests, especially for plants (Bonal et al., 2016; Nepstad et al., 2007; Phillips et al., 2010), little is known about how increased seasonal variations propagate beyond plant communities and affect microorganisms and animals, including those below the ground.

The belowground ecosystem compartment hosts a major part of terrestrial biodiversity and processes most of the primary production in terrestrial ecosystems, including tropical forests (Cebrian, 1999). Decomposers, from microorganisms to large invertebrates, are responsible for breaking down litter materials, releasing nutrients, and making them available for other living organisms and plants (Bardgett & van der Putten, 2014). Since temperature and water availability are among the key factors regulating soil biological activity, decomposer communities are sensitive to seasonal changes (Conant et al., 2011; Gomez et al., 2020; Liu et al., 2009; Taylor et al., 2004; Yu et al., 2019). For example, in a montane rainforest of Ecuador, precipitation and water content experimentally reduced to 10%–50% of its original value had dramatic consequences for microbial decomposer communities, with microbial biomass being reduced by 50% and microbial predators by as much as 90% (Krashevska et al., 2012). There are only a few studies that investigated the effects of seasonality on decomposer communities in tropical ecosystems. In the rainforests of China, Ecuador, and Mexico, seasonal changes in temperature and humidity have been reported to affect the diversity and composition of litter arthropods (Beng et al., 2018; Grimbacher et al., 2018; Jacquemin et al., 2016; Marín et al., 2016); however, in Brazil and Australia, arthropod diversity did not respond in a uniform way to seasonal dynamics (Grimbacher & Stork, 2009; Montine et al., 2014). As shown in climate experiments in the temperate zone, land use may modulate climatic effects on the abundance and diversity of soil organisms (Yin et al., 2019, 2020), but the aspect of seasonality has been poorly explored.

Across equatorial lowland regions, Southeast Asia is leading in deforestation rates and land‐use change to plantation monocultures in particular rubber and oil palm, which has implications on the climatic conditions not only locally but also globally (Sabajo et al., 2017). In Indonesia and Malaysia combined, oil palm monoculture covered more than 1.7 million ha in 2015 (Chong et al., 2017). Here, we build on the regional‐scale data collected in the dominating lowland ecosystems of Jambi province, Sumatra, Indonesia, that is, rainforest, and plantations of rubber and oil palm, in the framework of the multidisciplinary German‐Indonesian EFF or TS project (Drescher et al., 2016; Grass et al., 2020). Previous studies at our study sites have shown that canopy openness in plantations is approximately six times higher than in rainforest (Drescher et al., 2016). This is likely to affect local soil conditions since canopy openness is among the most important factor regulating the below‐canopy microclimate at our study sites (Camarretta et al., 2021; Meijide et al., 2018). The daily amplitude and magnitude of below‐canopy air temperature and air humidity have been reported to be larger in monocultures than in rainforest (Meijide et al., 2018). Shallow litter layer and root systems in plantations (Krashevska et al., 2015; Zuraidah et al., 2010), especially in oil palm, may additionally shift microclimatic conditions for soil organisms by changing evaporation and water uptake by plants. Moreover, management practices in oil palm and rubber plantations, such as weeding, herbicide application, and fertilization, impact microclimatic conditions through changes in understory plant cover, soil porosity, and water infiltration (Allen et al., 2015; Darras et al., 2019; Haruna et al., 2018). All these changes may contribute to the differences in variations in soil, microbial, and animal parameters between rainforest and plantations. Thus, in the present study, we investigated how land use and microclimatic seasonal variability affect soil parameters, microbial communities, and dominant groups of soil fauna. We surveyed rainforest and monocultures of oil palm and rubber on 12 distinct sites in four seasons, that is, four climatically different parts of the year, in 2017. Our main hypothesis was that seasonal variation of microbial and animal parameters is altered and more pronounced in plantations than in rainforest, with attenuated effect on animals in comparison to microbes because the latter respond more rapidly to environmental changes. Since microbes regulate the cycling and sequestration of carbon in soil, we specifically investigated microbial community indicators of carbon availability (Gram‐negative‐to‐Gram‐positive bacteria ratios) and cycling (fungal‐to‐bacterial ratios) (Fanin et al., 2019; Malik et al., 2016). Assuming that the soil layer is better protected against climatic variability than the surface litter layer, we further expected that seasonal variations in microbial and animal communities will be associated with changes in the vertical distribution of microbial and animal communities, especially in plantations. Finally, by assessing parallel seasonal changes in microclimate, we also aimed at investigating if associations between microclimate and decomposer communities depend on land use.

## 2 MATERIALS AND METHODS

### 2.1 Study area and sampling

The study took place in the tropical lowlands of southeast Sumatra, Indonesia (Drescher et al., 2016). Rainforest and monoculture plantations of rubber and oil palm were studied in the Harapan region of the Jambi province (1°55′40′′S, 103°15′33′′E; (Drescher et al., 2016). Rainforest used as a reference land use comprised primary degraded rainforest (Rembold et al., 2017). Rubber plantations comprised rubber (*Hevea brasiliensis* Muell. Arg.) monocultures with an average age of 16 years, while oil palm (*Elaeis guineensis* Jacq.) plantations comprised oil palm monocultures with an average age of 17 years. Each of the three land‐use types was replicated four times, resulting in 12 sampling sites, each with a 50 × 50 m sampling plot. Inside each sampling plot, one 5 × 5 m subplot was established (subplot “a” in Drescher et al., 2016).

We sampled each sampling site four times in 2017: March, June, September, and November (hereafter, referred to as “seasons”). The four seasons were assumed to cover the full range of seasonal variations in the region (Drescher et al., 2016). February and March of 2017 are rather wet, with rainfall almost every day, so sampling in March represents the end of the rainy season. April to mid‐June is characterized by frequent precipitation but also some days without precipitation, so sampling in June represents the wet to dry season transition period. Mid‐June to the beginning of September is characterized by two long periods of drought, only interrupted by a slightly wetter period in mid‐August, so sampling in September represents the peak of the dry season. From October to the end of the year, precipitation occurs frequently, with only a few days without precipitation, so sampling in November represents the start of the rainy season (Drescher et al., 2016).

For microbial and soil parameters, five cores were taken from the subplot to account for small‐scale spatial variation. Litter (L/F horizon) and upper mineral soil samples (A_h_ horizon, to a depth of 5 cm) were taken using a soil corer 5 cm in diameter. Litter and soil samples were pooled from each set of five cores to obtain two mixed samples per subplot (one for litter and one for soil). Seeds, twigs, roots, and coarse woody debris were removed by hand. Before analyses, the soil was sieved (4‐cm mesh) and litter was cut to pieces (ca. 1 cm). From these samples, water content, microbial biomass, pH, amount of litter and roots, carbon and nitrogen concentration of litter, phospholipid fatty acid (PLFA) markers and their ratios, as well as microbial biomass were analyzed (see below). To sample animals, one 16 × 16 cm sample was taken using a spade from each subplot (see *Animal abundance* below). Litter and upper mineral soil (to a depth of 5 cm) were processed separately. In total, 96 samples were taken and analyzed for soil parameters, microorganisms, and animals (3 land uses × 4 seasons × 2 layers × 4 replicates).

### 2.2 Meteorological measurements

A network of meteorological stations in the Harapan landscape was used to monitor below‐canopy microclimatic conditions in rainforest, rubber, and oil palm monoculture plantations (Meijide et al., 2018). In the center of each 50 × 50 m sampling plot, a meteorological station was established. Below‐canopy air temperature and relative humidity were measured at 2 m height above the ground surface with a thermohygrometer (Galltec+Mela, Bondorf, Germany) and soil temperature and soil moisture at 30 cm depth below the ground surface with a Trime‐Pico 32 soil probe (IMKO, Ettlingen, Germany) at an interval of one measurement per hour. UIT LogTrans 16‐GPRS data loggers (UIT, Dresden, Germany) were used to record the data. In addition to the below‐canopy microclimatic conditions, we used precipitation measurements from three open‐area meteorological stations in the Harapan region located at the same elevation. At these stations, precipitation was measured at 1.5‐m height above the ground surface with two tipping bucket precipitation gauges at each station (Thies Clima, Göttingen, Germany) and stored on a DL16 data logger (Thies Clima).

### 2.3 Soil and microbial parameters

For testing the impact of seasonality on soil parameters, part of the litter and soil material was dried at 65°C for 72 h, milled, and analyzed for total C and N concentrations using an elemental analyzer (Carlo Erba; Milan, Italy). Soil pH (CaCl_2_) was measured using a digital pH meter (Greisinger GPHR 1400A, Regenstauf). The amount of litter and water content of litter and soil were determined gravimetrically from 16 × 16 cm samples for the animal extraction (Table 1). To measure water content, substrates were weighed fresh and air‐dried (50°C for 1 week).

**TABLE 1:** Measured parameters for soil and litter, microorganisms, and animal taxa. Only dominant PLFA biomarkers and animal groups present in at least 60% of the samples were analyzed

Parameter	Method	Units	Description
**Soil and litter**			
Litter amount	Gravimetry	g litter in a 16 × 16 cm area	The buffering cover of the soil, habitat, and resource for microbes and fauna (Fujii et al., 2020)
Roots	Gravimetry	g fresh fine roots (<4 mm in diameter) in g^−1^ dry weight of soil	Reflect potential supply of labile carbon to soil organisms (Eisenhauer et al., 2017; Pollierer et al., 2007)
Water content	Gravimetry	wet weight (proportion of dry weight)	Optimum moisture supports the high activity of soil organisms (Bahram et al., 2018; Bickel & Or, 2020; Gomez et al., 2020; Krashevska et al., 2012)
pH(CaCl_2_)	Digital pH meter	–	Proxy for substrate acidity affects the composition of soil communities, such as fungi‐to‐bacteria ratio (Bahram et al., 2018; Johannes & Erland, 2009)
C and N concentrations	Elemental analyzer	total C (%); total N (%)	Proxy for the quality of food resources for microbes and fauna
**Microorganisms**			
Basal respiration	Automated respirometer system	μg O_2_ h^−1^ g^−1^ soil dry weight	Represents the total microbial activity (Scheu, 1992)
Microbial biomass	Automated respirometer system	C_mic_; μg g^−1^ dry weight	Represents the total living microbial biomass (Scheu, 1992)
Gram‐negative bacteria (GN bacteria)	PLFAs: 16:1ω7, cy17:0 and cy19:0	nmol g^−1^ dry weight	Relative markers of Gram‐negative bacteria, the sum represents the active community of Gram‐negative bacteria (Zelles, 1997, 1999). Microbial decomposer (use more plant‐derived C sources; Kramer & Gleixner, 2008); N‐fixators, food for animals and protists.
Gram‐positive bacteria (GP bacteria)	PLFAs: i15:0, a15:0, i16:0, and i17:0	nmol g^−1^ dry weight	Relative markers of Gram‐positive bacteria, the sum represents the active community of Gram‐positive bacteria (Zelles, 1997, 1999). Microbial decomposer (use more organic matter derived C sources; Kramer & Gleixner, 2008); food for animals and protists.
Fungi	PLFA: 18:2ω6,9	nmol g^−1^ dry weight	Relative marker of saprophytic fungi (Frostegard & Baath, 1996; Ruess & Chamberlain, 2010). Decomposers, food for animals and protists.
Gram‐positive‐to‐Gram‐negative bacteria ratio (GP:GN ratio)	GP:GN bacterial PLFAs	ratio	Relative indicator of carbon availability; high values indicate lower availability (Fanin et al., 2019)
Fungi‐to‐bacteria ratio (F:B ratio)	Fungal‐to‐bacterial PLFAs	ratio	Relative indicator of carbon cycling; high values indicate slower cycling and greater C storage potential (Malik et al., 2016)
**Animals**			
Oribatida	Visual sorting	individuals in a 16 × 16 cm sample	Microdecomposers, feeding on detritus and microorganisms
Collembola	Visual sorting	individuals in a 16 × 16 cm sample	Microdecomposers, feeding on detritus and microorganisms
Mesostigmata	Visual sorting	individuals in a 16 × 16 cm sample	Micropredators, feeding on microdecomposers and nematodes
Symphyla	Visual sorting	individuals in a 16 × 16 cm sample	Microdecomposers, feeding on microorganisms
Diptera	Visual sorting	individuals in a 16 × 16 cm sample	Mixed functional role (include detritivores, microbivores, predators, and herbivores)
Formicidae	Visual sorting	individuals in a 16 × 16 cm sample	Omnivores with diverse food resources
Psocoptera	Visual sorting	individuals in a 16 × 16 cm sample	Microdecomposers, feeding on detritus and microorganisms
Coleoptera	Visual sorting	individuals in a 16 × 16 cm sample	Mixed functional role (include detritivores, microbivores, predators, and herbivores)
Total soil animal metabolism	Visual sorting and metabolic regressions	Joule per hour per 16 × 16 cm area	Proxy for the total feeding activity of soil animals

Basal respiration and microbial biomass in litter and soil were determined by measuring oxygen (O_2_) consumption with an automated respirometer system (Scheu, 1992). Microbial basal respiration of moist field samples (1 g L/F material cut to pieces <25 mm^2^ and 5 g A_h_ material sieved <2 mm) was measured at 22°C; the mean O_2_ consumption during hours 10–20 after attachment to the respirometer was used. Microbial biomass carbon was assessed by measuring the maximum initial respiratory response (MIRR) after glucose addition at 22°C and calculated as 38× MIRR (Anderson & Domsch, 1978; Beck et al., 1997; Joergensen & Scheu, 1999). Glucose (80 and 40 mg g^−1^ dry wt for L/F horizon and A_h_ horizon, respectively) was added as an aqueous solution adjusting the soil water content to 80%–90% of the water holding capacity of the litter and soil materials. The mean of the three lowest measurements during the first 10 h after glucose addition was taken as the MIRR (for details see Table 1).

Major microbial groups in litter and soil, such as bacteria and fungi, were analyzed by fatty acids analysis (Frostegård et al., 2011). For measuring phospholipid fatty acids (PLFAs), 1 g L/F material and 2 g A_h_ material were extracted following the procedure of Frostegård et al. (1993). Individual PLFA biomarkers and their ratios were used to represent changes in microbial communities and indicator functions (Table 1). The fungal‐to‐bacterial PLFA (F:B) ratio is related to carbon cycling with higher ratios reflecting slower cycling (Malik et al., 2016). The Gram‐negative‐to‐Gram‐positive PLFA (GN:GP) ratio is related to carbon availability with higher ratios reflecting lower availability (Fanin et al., 2019).

### 2.4 Animal abundance

Soil animals were extracted from the litter and soil samples using a heat gradient from 40–50°C above to 15°C below the sample (Kempson et al., 1963), collected in dimethylene glycol ‐ water solution (1:1), and transferred into 70% ethanol. All animals were counted and sorted to broad taxonomic groups (orders and families; Table 1) under a dissecting microscope. We used previous data sets on metabolic rates of soil fauna from the rainforest, oil palm, and rubber monocultures at the same sampling sites (Potapov et al., 2019) to calculate average land use‐specific per group metabolic rates. We multiplied average metabolic rates by the numeric abundance of taxonomic groups and summed them up to calculate total soil animal community metabolism per square meter for each sampling site. Animal community metabolism representing total animal activity was compared to basal respiration representing total microbial activity.

### 2.5 Data analysis

For animals and fatty acids, we selected groups that were present on more than 60% of sites because groups that were found on fewer sites likely to be undersampled and corresponding models poorly described the data. All fatty acid proportions and proportions of different microbial groups were logit‐transformed prior to the analysis for normal distribution approximation and variance stabilization (Warton & Hui, 2011).

To test the effect of seasonality on different components of the decomposer system, we applied linear mixed‐effects modeling (LME) as implemented in the *lme4* package (Bates et al., 2015). “Season”, “Land use”, “Layer,” and their pairwise interactions were added as fixed factors and “Plot” and “Sample” (i.e., plot at particular season) as random intercepts (*n* = 96). Data distribution for each parameter was visually checked and the following data distributions were fitted to the initial models according to the data type and distribution: (1) for animal counts, we used generalized models with Poisson distribution; (2) for logit‐transformed fatty acid data and soil parameters, we used Gaussian distribution; (3) for the total animal community metabolism data, we used Gaussian distribution after log‐transformation. Before fitting a model, we controlled for outliers in each parameter with Rosner's generalized extreme Studentized deviate test using *rosnerTest* from the *EnvStats* package (Millard, 2013). In total, 8 outliers out of 2640 observations were detected (0.3%). To keep all replicates, we corrected the offset from the closest non‐outlier value for each outlier by −80% (an arbitrary value, but the selection is unlikely to affect any results). Corrected data included sampling or technical analysis biases, for example, a sample with extremely high local density of Formicidae (an ant nest was sampled) or a waterlogged sample with high water content. This was done to improve the fit of the models for other observations. After fitting the initial models for each response variable, we checked model residuals for heteroscedasticity using *leveneTest* from the *car* package (Fox & Weisberg, 2019). If heteroscedasticity was detected among land uses or layers, we re‐fitted the model using generalized least squares as implemented in the *nlme* package (GLS) (Pinheiro et al., 2020). This allowed us to explicitly account for different scales of data variation in soil and litter, or in the different land uses (Zuur et al., 2009). Effect significance in the final model was evaluated with Wald chi‐square analysis of variance using *Anova* from the *car* package.

For other analyses, we normalized and scaled data to make it comparable across all variables. Skewed data, that is, animal counts and total animal community metabolism were log‐transformed (zeros were included by adding minimum value across all non‐zero observations). After that all variables were scaled and centered around zero; we used z‐standardization, that is, subtracted means and divided all data by its standard deviations.

To test if the magnitude of seasonal variations is higher in transformed ecosystems than in rainforest and in the litter than in soil, we compared seasonal coefficients of variation in all studied parameters using the scaled data. First, we calculated coefficients of variation (CVs) across four seasons for each parameter in each plot and layer (*n* = 4 per plot). We further compared average CVs across plots in the rainforest with those in rubber and oil palm using pairwise comparisons with *t.test* with Welch approximation for the degrees of freedom.

To study how the association of climatic and soil variables depends on the land use and the time frame at which the climatic data were measured, we applied non‐metric multidimensional scaling (NMDS) as implemented in the *vegan* package (Oksanen et al., 2020). We used averaged values across plots for each land use since there were some technical gaps in climatic data on individual plots. We further included dominant animal taxonomic groups, total animal metabolism, individual fatty acid concentrations, and soil parameters in the analysis (Table 1). All variables were controlled for outliers, transformed, and scaled prior to the analysis as described above. NMDS was run separately in each of the three land uses (k was set to three to ensure the stress value below 0.1). The effect of climatic variables was assessed by averaging below‐canopy air humidity and soil moisture over a period of 3, 13, and 28 days before the sampling date and fitting them onto the NMDS ordination using *envfit* from the *vegan* package.

## 3 RESULTS

### 3.1 Climate

**FIGURE 1:**
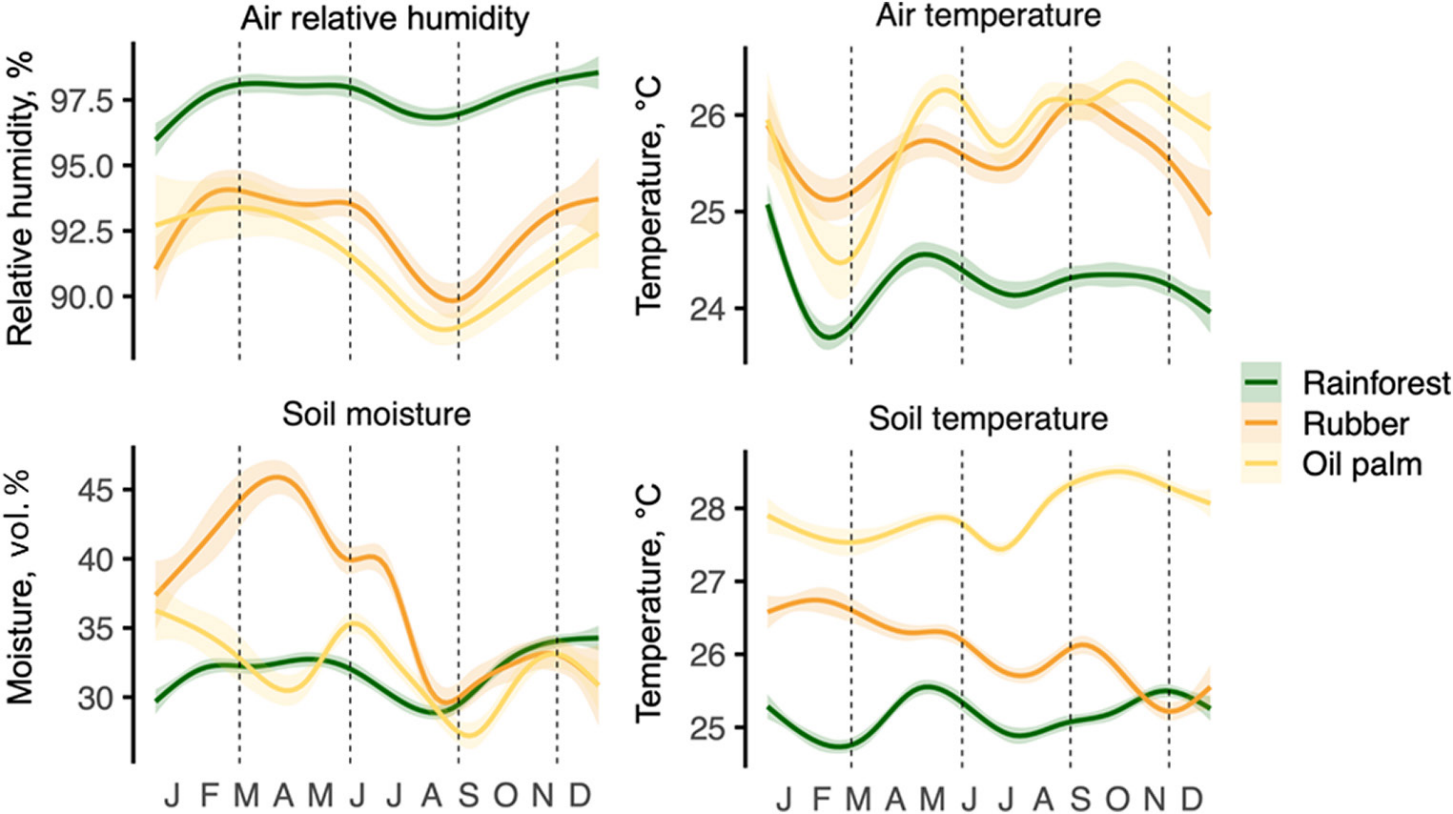
Seasonal variations in below‐canopy air relative humidity and temperature, and in soil moisture and temperature (30 cm depth) at the study sites in 2017. Local polynomial regression smoothers with 95% confidence intervals are shown. Different land uses are shown with colors: rainforest (green), rubber (orange), and oil palm (yellow). Vertical dashed lines indicate the four sampling dates: March, June, September, and November

Below‐canopy air relative humidity, air temperature, soil moisture, and soil temperature followed a distinct seasonal pattern, with the lowest air humidity and soil moisture in all land uses in August and September (the peak dry season; Figure 1, Table S1). The air temperature was lowest in February and March (the peak rainy season). Across the year, the rainforest had about 5% higher air relative humidity and about 2°C lower temperature of both air and soil than plantations. Soil moisture was similar across land uses, except for high moisture in March–April in some rubber plots (Figure 1, Table S1). The magnitude of seasonal variation of air humidity was 16%–20% higher in both monoculture plantations than in rainforest, while that of air temperature was 8% higher in oil palm only (Figure S1).

### 3.2 Decomposer system

**FIGURE 2:**
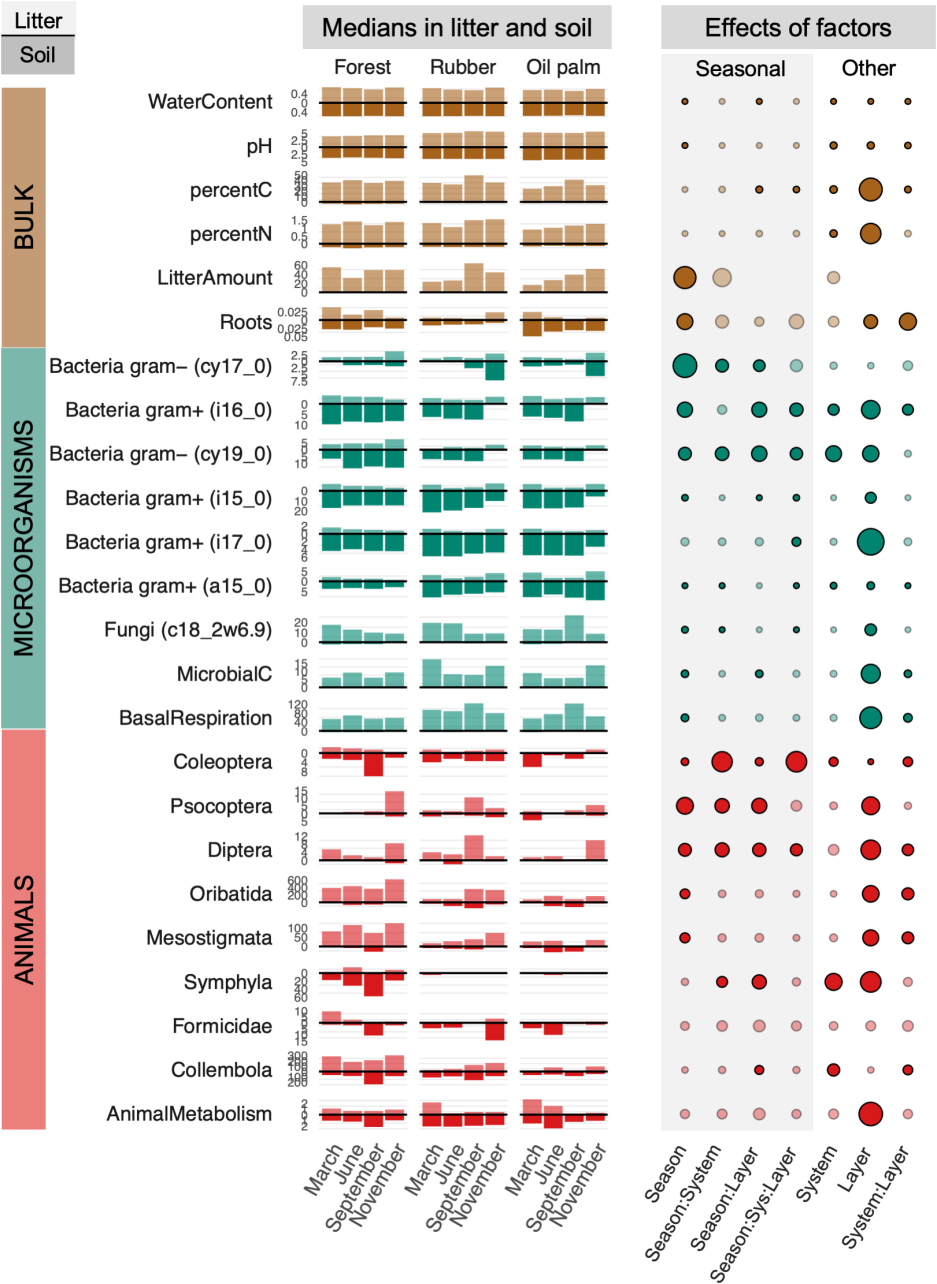
Seasonal variations in parameters for soil and litter (bulk), microorganisms, and animal taxa (animals) in rainforest, rubber, and oil palm plantations. Histograms show medians in the litter (light color, above the line) and soil (full color, below the line) for each parameter in each Season and Land use (Sys). Measurement units are given in Table 1. The bubble diagram shows the results of linear mixed‐effects modeling. Bubble sizes are proportional to the chi‐square of the corresponding factor effects; dark circled bubbles indicate significant effects

Season, either directly or in interaction with Land use or Layer, significantly affected all the measured parameters, except nitrogen concentration in litter and soil, number of Formicidae, and total animal metabolism (Figure 2; for mean values and standard deviations see Tables S2 and S3; for units Table 1; for statistical analysis Table S5). Most of the litter and soil parameters (pH, litter amount, and root biomass) were affected by Season independently of Land use and Layer, whereas water content varied interactively with Season and Layer (reduction in water content by 4% in soil and 13% in litter in September). Vertical distribution of carbon concentration, but not that of nitrogen, varied with Season and Land use (Tables S2 and S3).

Almost all microbial parameters were affected directly by the Season (Figure 2). However, the effect of Season varied with Land use and Layer for all bacterial and fungal biomarkers, with bacterial biomarkers cy19:0 (Gram−) and i16:0 (Gram+) showing the strongest response (significant Season × Land use × Layer interaction; Figures 2, S2). In 7 out of 11 microbial parameters, the Season × Layer interaction was significant, whereas the Season × Land use interaction was only significant in 3 out of 11 microbial parameters. The microbial parameters (basal respiration and microbial carbon) changed similarly with season across land uses, but the vertical distribution of microbial carbon was modified by Land use and Season.

**FIGURE 3:**
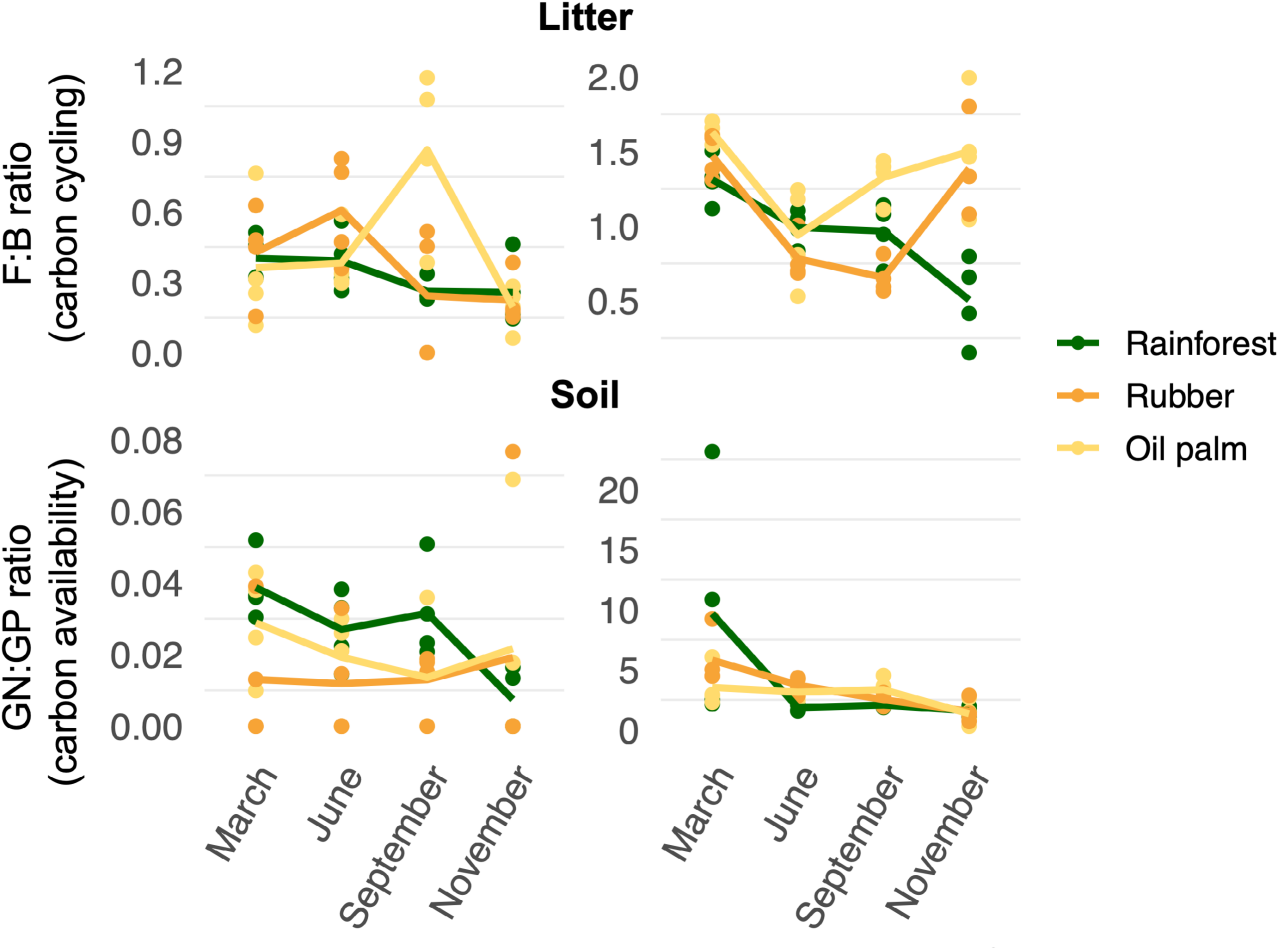
Seasonal variations in the fungi‐to‐bacteria (F:B) ratio and Gram‐negative‐to‐Gram‐positive (GN:GP) ratio in litter (upper panel) and soil (lower panel) of different land uses (rainforest, rubber, and oil palm plantations)

All microbial biomarkers, except PLFA i17:0, varied with Season (Figure 2; Tables S2, S3). In 2 Gram‐negative bacterial PLFAs (cy17:0, cy19:0) and 1 Gram‐positive bacterial PLFA (a15:0) as well as the fungal PLFA (18:2ω6,9), the Season × Land use interaction was significant (Figure 2, Tables S2 and S3). Further, the vertical distribution between litter and soil of all microbial biomarkers except PLFA cy17:0 varied with Season and Land use (significant Season × Land use × Layer interaction; Figure 2, Tables S2 and S3). Generally, the F:B ratio was 91% higher in litter than in soil, but it varied interactively with Land use, Season, and Layer (significant Season × Land use × Layer interaction; Figure 3, Tables S2 and S3). In litter of all land uses, the F:B ratio was lowest in November; however, in litter of rainforest, it continuously decreased from March to November (−36%), whereas in litter of rubber, it was highest in June (48% higher than in November) and in oil palm in September (77% higher than in November) (Figure 3, Tables S2 and S3). Similar to litter, in rainforest soil, the F:B ratio decreased from March to November (−82%), but not in plantations. In soil of rubber, it was highest in November and lowest in June (−37%) and in oil palm, it was highest in March and lowest in September (−52%) (Figure 3, Tables S2, S3 and S5). In contrast to the F:B ratio, the GN:GP ratio was 66% higher in soil than in litter, but it also varied interactively way with Land use, Season, and Layer (significant Season × Land use × Layer interaction; Figure 3, Tables S2, S3 and S5).

In litter of all land uses, the GN:GP ratio was highest in March; however, in litter of rainforest, it continuously decreased from March to November (−61%), whereas in litter of rubber, it was lowest in September (55% lower than in March) and in oil palm in June (42% lower than in March) (Figure 3, Tables S2 and S3). Similar to litter, in soil, the GN:GP ratio was highest in March and it continuously decreased from March to November in soil of rainforest (−83%), rubber (−73%), and oil palm plantations (−62%) (Figure 3, Tables S2 and S3).

Among animal groups, Psocoptera, Diptera, Oribatida, and Mesostigmata were 30–80% more abundant in September and November, as compared to March and June, in each land use (Season effect, Figure 2, Tables S2 and S3). In Psocoptera, Diptera and Symphyla seasonal changes in abundance varied with Land use (Figure 2, significant Season × Land use interaction, Tables S2 and S3). The vertical distribution between litter and soil varied with Season in five out of nine animal groups and this effect varied with Land use for Diptera and Coleoptera (Figure 2, significant Season × Land use × Layer interaction, Tables S2 and S3). Total animal metabolism generally did not vary consistently with Season and Land use but was higher in soil than in litter at most sampling dates (Figure 2, Layer effect, Tables S2 and S3).

### 3.3 The magnitude of seasonal variations and correlations with climate

**FIGURE 4:**
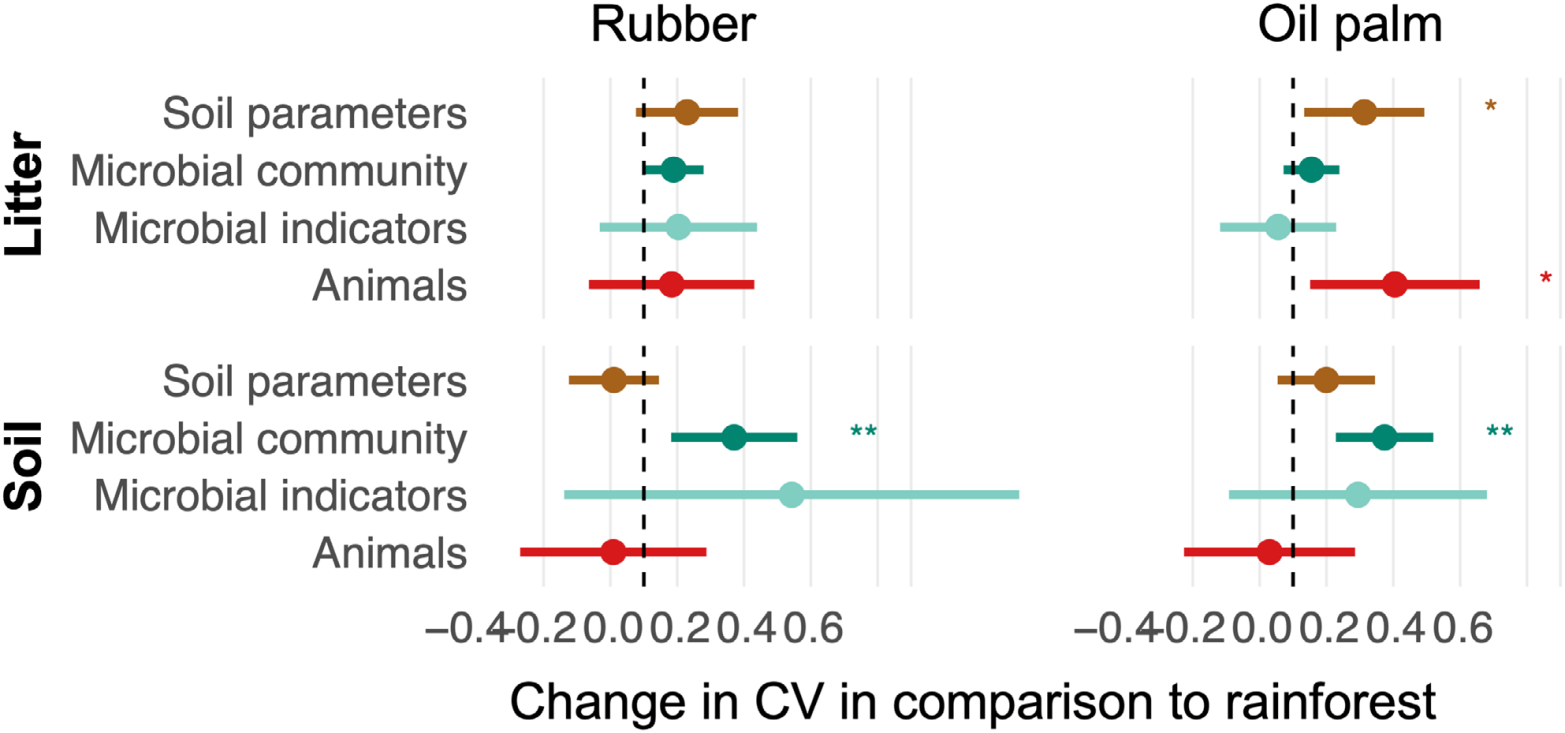
Differences in the magnitude of seasonal variations in soil parameters, microbial community, microbial indicators, and, density of animal groups in litter (upper panel) and soil (lower panel) between rainforest and rubber (left) and rainforest and oil palm plantations (right). Coefficients of variation (CV) of samples are taken at four seasons. Confidence intervals that do not cross the (dashed) zero line indicate significant differences to rainforest

In both plantations, seasonal variations in microbial community indicators were almost 40% higher than in rainforest (Figures 4 and S2). In addition, in litter of oil palm plantations, seasonal variations in animal abundance were up to 40% higher than in rainforest (mostly driven by Collembola, Mesostigmata, and Symphyla), and in soil parameters, it was almost 35% higher (mostly driven by water content, carbon concentration, and roots biomass; Figures 4 and S2).

**FIGURE 5:**
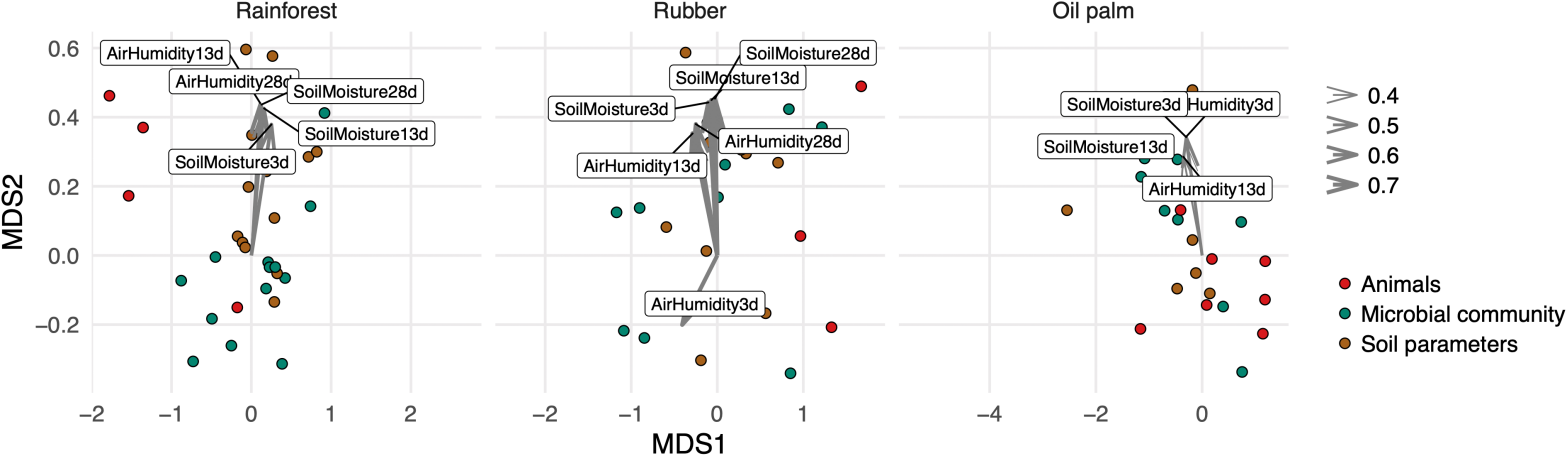
Non‐metric multidimensional scaling shows the association of soil parameters (brown), microbial parameters (green), and animal groups (red) with climate variables at different temporal scales. Parameters in litter and soil were bulked. NMDS was constructed using three axes; stress values were 0.081–0.095. Climate variables were averaged for the period of 3, 13, and 28 days before taking the samples. Only significant associations are shown, arrow thickness reflects the explanatory power (*R* ^2^)

Soil moisture averaged for 3 days before the sampling was significantly associated with parameters of the decomposer system in rubber and oil palm plantations (*R* ^2^ = 0.44–0.67, *p* < .010) but not in rainforest (*R* ^2^ = 0.07, *p* = .589, Figure 5). By contrast, soil moisture and air humidity averaged for 28 days before the sampling, were not associated with parameters of the decomposer system in oil palm plantations (Table S4).

## 4 DISCUSSION

Here, we analyzed complex changes in the decomposer system associated with seasonal variations in climatic conditions under tropical land‐use change. The majority of the measured litter and soil, microbial, and animal parameters (20 out of 26) changed with the season. Land use shifted these seasonal changes in 13 parameters, either directly or by modifying the vertical distribution of the given parameter. Further, the magnitude of the seasonal variations in microbial parameters in soil of rubber and oil palm monocultures considerably exceeded that in rainforest (by almost 40%). These changes reflect the higher sensitivity of the decomposer system of plantations to short‐term (3 days) variations in microclimatic conditions compared to rainforest.

Our main hypothesis was that seasonal variations in microbial and animal parameters (their maxima and minima) shift in time and are more pronounced in plantations than in rainforest. This was supported especially for microorganisms, as seasonal changes in the majority of microbial parameters were affected by land use. Furthermore, the F:B PLFA ratio as indicator of carbon sequestration (Malik et al., 2016) showed higher seasonal variation in plantations than in rainforest, where it was similar throughout the year. Higher GN:GP bacterial PLFA ratios in plantations in most of the seasons, especially in litter, indicate lower C availability and thereby lower ecosystem productivity in plantations than in rainforest (Fanin et al., 2019). This may indicate higher sensitivity of microorganisms in plantations to seasonal climatic changes than in rainforest. However, plantation management such as herbicide and fertilizer applications may also have contributed to the observed differences. Among soil animals, seasonal variations in rainforest were less pronounced than in plantations in Psocoptera, Diptera, and Coleoptera but not in other taxa. This is in line with our expectation that soil microorganisms more sensitively and more quickly respond to environmental changes than soil animals. This may be due to higher growth rates of microorganisms than animals and higher dependency on water availability as well as seasonal changes in litter and soil parameters, for example, pH (Bahram et al., 2018; Bickel & Or, 2020). Surprisingly, only a few studies investigated seasonal changes in soil microorganisms in different land uses (Lan et al., 2021; Lepcha & Devi, 2020), whereas several studies investigated seasonal changes in soil animals (Grimbacher et al., 2018; Grimbacher & Stork, 2009; Montine et al., 2014), but, to the best of our knowledge, no studies exist for Southeast Asia (but see Beng et al., 2018). Similar to our study, the abundance of litter‐dwelling Coleoptera in tropical rainforests of Australia varied with season (Grimbacher & Stork, 2009). However, in contrast to Coleoptera, Formicidae did not show any changes with seasonality. This is in line with the results on epigaeic ants from a Brazilian Atlantic rainforest but contrasts findings from other tropical forests (Grimbacher et al., 2018; Jacquemin et al., 2016; Montine et al., 2014). Also, microarthropods, such as Collembola and Oribatida, did not show clear seasonal changes in the different land uses, contrasting results from the tropical forest and rubber plantations in China (Beng et al., 2018). In general, in our study, changes in seasonal dynamics with changes in land use were more pronounced in soil macrofauna, that is, Coleoptera, Psocoptera, and Diptera (except Formicidae) than in microarthropods such as Oribatida, Mesostigmata, and Collembola (except Symphyla).

According to our second hypothesis, seasonal variations modify the vertical distribution of microbial and animal communities, especially in plantations. Although the results of this study generally support this hypothesis, the results also suggest that the effects of seasonal climatic variations on soil microorganisms are more pronounced than on soil animals. In microorganisms, the effect of season and land use were different in litter and soil in 8 of 11 microbial parameters and in soil animals, it was in 2 out of 11 parameters. Also, both indicators of carbon availability (F:B and GP:GN ratios) had significant threefold interactions, suggesting that carbon use across soil and litter is less stable, especially in plantations. Previous studies have also reported that in addition to land use and seasons, soil depth affects microorganisms (Lepcha & Devi, 2020; Seuradge et al., 2017). However, there is a lack of knowledge on differences in microbial communities between litter and soil layers, as most studies investigating depth profiles focused on mineral soil and ignored the litter layer (Fierer et al., 2003; Ko et al., 2017). This is unfortunate as saprotrophic microorganisms are concentrated in the litter layer rather than the soil, especially in tropical regions including our study sites (Krashevska et al., 2015). In animals, land use modified seasonal changes in the vertical distribution of Diptera and Coleoptera. This conforms to previous studies showing the sensitivity of Diptera and Coleoptera to seasonal climatic changes, especially in plantations (Beng et al., 2018). The changes in abundance and depth distribution in these taxa are likely related to their life cycle and larval stage in soil. Many Diptera and Coleoptera species are associated with the litter layer and the shallow litter layer and more pronounced variations in environmental factors in plantations may render them susceptible to seasonal climatic variations. In fact, the litter layer of tropical forests is an important habitat for soil organisms, and many soil animal taxa suffer from shallower litter layers in plantations (Beng et al., 2018; Krashevska et al., 2015; Susanti et al., 2021). Further, the amount of litter changes considerably with seasons and these changes are most pronounced in plantations, especially in oil palm, with the amount of litter being lowest in March and June following management practices, whereas in rainforest, the amount of litter stays more constant throughout the year. Unexpectedly, Formicidae, which have been shown previously to respond to seasonal changes in climate by changing their vertical distribution in soil (Jacquemin et al., 2016), did not show any variation in vertical distribution with the season or land use at our study sites. This, however, may reflect an insufficient sampling of this group due to the patchy distribution of ant colonies (Rizqulloh et al., 2021).

For identifying environmental factors responsible for the observed changes in seasonal variations in microbial and animal parameters, we correlated short‐ and long‐term fluctuations in environmental factors with microbial and animal parameters. The results indicated that in plantations, but not in the rainforest, fluctuations in the decomposer system were driven by short‐term meteorological variability. This highlights the sensitivity of the decomposer system in plantations to variations in climate which presumably is due to reduced buffering of changes in environmental factors due to the shallow litter layer in plantations. Earlier studies in central Amazonia also pointed to the sensitivity of soil microorganisms and mesofauna to changes in microclimatic conditions (Kurzatkowski et al., 2004; Martius et al., 2004). Overall, these findings indicate that the decomposer system in plantations is little buffered against short‐term microclimatic fluctuations. In particular, microorganisms and animals in the upper biologically most active soil layers may suffer from these fluctuations. Multiple groups of soil microorganisms and animals together support soil functioning (Wagg et al., 2014). The differential responses of different groups to the changes in microclimate may decouple established soil interaction networks that develop over long time periods and sustain the efficiency of carbon use and sequestration (Morriën et al., 2017). In this context, the observed changes in microbial parameters, related to basic biogeochemical processes, and macrofauna abundances, related to animal engineering of soils, may have complex compromising effects on soil functioning and the services soils provide. This destabilization of the decomposer system is especially worrying in face of the increased frequency of extreme climatic events under global change affecting above‐belowground ecosystem functioning.

To conclude, we investigated for the first time seasonal changes in the decomposer system including a wide range of soil, microbial, and animal parameters in rainforest and major agricultural replacement systems in South East Asia, a focal area of land‐use change in the tropics. Soil, microbial, and animal parameters including indicators of the carbon cycle varied with the season in both rainforests, and oil palm and rubber plantations. Indicators of the structure and functioning of microbial communities varied strongly with the season in plantations but not in rainforest, and this was also true for indicators of animal communities but only in litter. Seasonal variations in air relative humidity over a time window of 3 days were associated with variations in the decomposer system in plantations, but not in rainforest pointing toward the loss of buffering capacity of the belowground system with the conversion of rainforest into plantations. Reduced buffering in plantations was associated with reduced litter layer opening the perspective for management practices mitigating the reduced buffering capacity of the belowground system, for example, mulching practices (Formaglio et al., 2021; Tao et al., 2018). Our results further suggest that microorganisms are a sensitive indicator of climatic changes in transformed land uses. Overall, our findings suggest that land‐use change shifts and magnifies seasonal variations of the belowground ecosystem, especially the structure and functioning of microbial communities, with potentially major ramifications for the services they provide such as carbon and nutrient cycling. These ramifications ultimately may compromise the stability of tropical ecosystems in particular in face of global climate and land‐use change.

## AUTHOR CONTRIBUTIONS

**Valentyna Krashevska:** Conceptualization (lead); data curation (lead); formal analysis (equal); funding acquisition (equal); investigation (lead); methodology (lead); project administration (lead); resources (lead); supervision (lead); visualization (equal); writing – original draft (lead); writing – review and editing (lead). **Christian Stiegler:** Data curation (equal); formal analysis (equal); investigation (equal); resources (equal); writing – original draft (equal); writing – review and editing (equal). **Tania June:** Data curation (supporting); methodology (supporting); project administration (equal); resources (supporting); writing – review and editing (supporting). **Rahayu Widyastuti:** Data curation (equal); project administration (equal); resources (equal); writing – review and editing (equal). **Alexander Knohl:** Data curation (equal); funding acquisition (equal); writing – review and editing (equal). **Stefan Scheu:** Conceptualization (equal); funding acquisition (lead); supervision (equal); writing – review and editing (equal). **Anton Potapov:** Conceptualization (equal); data curation (equal); formal analysis (lead); funding acquisition (equal); investigation (equal); methodology (equal); project administration (equal); resources (equal); software (lead); supervision (equal); validation (equal); visualization (lead); writing – original draft (equal); writing – review and editing (equal).

## CONFLICT OF INTEREST

The authors declare that they have no known competing financial interests or personal relationships that could have appeared to influence the work reported in this article.

## Supporting information


Figures S1‐S2

Tables S1‐S5

## Data Availability

All data are available as electronic supplementary material.

## References

[ece39020-bib-0001] Allen, K. , Corre, M. D. , Tjoa, A. , & Veldkamp, E. (2015). Soil nitrogen‐cycling responses to conversion of lowland forests to oil palm and rubber plantations in Sumatra, Indonesia. PLoS One, 10(7), e0133325. 10.1371/journal.pone.0133325 26222690 PMC4519237

[ece39020-bib-0002] Anderson, J. P. E. , & Domsch, K. H. (1978). A physiological method for the quantitative measurement of microbial biomass in soils. Soil Biology and Biochemistry, 10(3), 215–221. 10.1016/0038-0717(78)90099-8

[ece39020-bib-0003] Bahram, M. , Hildebrand, F. , Forslund, S. K. , Anderson, J. L. , Soudzilovskaia, N. A. , Bodegom, P. M. , Bengtsson‐Palme, J. , Anslan, S. , Coelho, L. P. , Harend, H. , Huerta‐Cepas, J. , Medema, M. H. , Maltz, M. R. , Mundra, S. , Olsson, P. A. , Pent, M. , Põlme, S. , Sunagawa, S. , Ryberg, M. , … Bork, P. (2018). Structure and function of the global topsoil microbiome. Nature, 560(7717), 233–237. 10.1038/s41586-018-0386-6 30069051

[ece39020-bib-0004] Bardgett, R. D. , & van der Putten, W. H. (2014). Belowground biodiversity and ecosystem functioning. Nature, 515(7528), 505–511. 10.1038/nature13855 25428498

[ece39020-bib-0005] Bates, D. , Mächler, M. , Bolker, B. , & Walker, S. (2015). Fitting linear mixed‐effects models using lme4. Journal of Statistical Software, 67(1), 1–48.

[ece39020-bib-0006] Beck, T. , Joergensen, R. G. , Kandeler, E. , Makeshin, E. , Nuss, E. , Oberholzer, H. R. , & Scheu, S. (1997). An inter‐laboratory comparison of ten different ways of measuring soil microbial biomass C. Soil Biology & Biochemistry, 29(7), 1023–1032. 10.1016/S0038-0717(97)00030-8

[ece39020-bib-0007] Beng, K. C. , Corlett, R. T. , & Tomlinson, K. W. (2018). Seasonal changes in the diversity and composition of the litter fauna in native forests and rubber plantations. Scientific Reports, 8(1), 10232. 10.1038/s41598-018-28,603-7 29980785 PMC6035245

[ece39020-bib-0008] Bickel, S. , & Or, D. (2020). Soil bacterial diversity mediated by microscale aqueous‐phase processes across biomes. Nature Communications, 11(1), 116. 10.1038/s41467-019-13,966-w PMC6949233 31913270

[ece39020-bib-0009] Bonal, D. , Burban, B. , Stahl, C. , Wagner, F. , & Hérault, B. (2016). The response of tropical rainforests to drought—Lessons from recent research and future prospects. Annals of Forest Science, 73(1), 27–44. 10.1007/s13595-015-0522-5 27069374 PMC4810888

[ece39020-bib-0010] Cai, W. , Wang, G. , Dewitte, B. , Wu, L. , Santoso, A. , Takahashi, K. , Yang, Y. , Carréric, A. , & McPhaden, M. J. (2018). Increased variability of eastern Pacific El Niño under greenhouse warming. Nature, 564(7735), 201–206. 10.1038/s41586-018-0776-9 30542166

[ece39020-bib-0011] Camarretta, N. , Ehbrecht, M. , Seidel, D. , Wenzel, A. , Zuhdi, M. , Merk, M. S. , Schlund, M. , Erasmi, S. , & Knohl, A. (2021). Using airborne laser scanning to characterize land‐use Systems in a Tropical Landscape Based on vegetation structural metrics. Remote Sensing, 13(23), 4794. 10.3390/rs13234794

[ece39020-bib-0012] Cebrian, J. (1999). Patterns in the fate of production in plant communities. The American Naturalist, 154(4), 449–468. 10.1086/303244 10523491

[ece39020-bib-0013] Chong, K. L. , Kanniah, K. D. , Pohl, C. , & Tan, K. P. (2017). A review of remote sensing applications for oil palm studies. Geo‐Spatial Information Science, 20(2), 184–200. 10.1080/10095020.2017.1337317

[ece39020-bib-0014] Conant, R. T. , Ryan, M. G. , Ågren, G. I. , Birge, H. E. , Davidson, E. a., Eliasson, P. E. , Evans, S. E. , Frey, S. D. , Giardina, C. P. , Hopkins, F. M. , Hyvönen, R. , Kirschbaum, M. U. F. , Lavallee, J. M. , Leifeld, J. , Parton, W. J. , Megan Steinweg, J. , Wallenstein, M. D. , Martin Wetterstedt, J. Å. , & Bradford, M. a. (2011). Temperature and soil organic matter decomposition rates ‐ synthesis of current knowledge and a way forward. Global Change Biology, 17(11), 3392–3404. 10.1111/j.1365-2486.2011.02496.x

[ece39020-bib-0015] Darras, K. F. A. , Corre, M. D. , Formaglio, G. , Tjoa, A. , Potapov, A. , Brambach, F. , Sibhatu, K. T. , Grass, I. , Rubiano, A. A. , Buchori, D. , Drescher, J. , Fardiansah, R. , Hölscher, D. , Irawan, B. , Kneib, T. , Krashevska, V. , Krause, A. , Kreft, H. , Li, K. , … Veldkamp, E. (2019). Reducing fertilizer and avoiding herbicides in oil palm plantations—Ecological and economic valuations. Frontiers in Forests and Global Change, 2, 65. 10.3389/ffgc.2019.00065

[ece39020-bib-0016] Drescher, J. , Rembold, K. , Allen, K. , Beckschäfer, P. , Buchori, D. , Clough, Y. , Faust, H. , Fauzi, A. M. , Gunawan, D. , Hertel, D. , Irawan, B. , Jaya, I. N. S. , Klarner, B. , Kleinn, C. , Knohl, A. , Kotowska, M. M. , Krashevska, V. , Krishna, V. , Leuschner, C. , … Scheu, S. (2016). Ecological and socio‐economic functions across tropical land use systems after rainforest conversion. Philosophical Transactions of the Royal Society B: Biological Sciences, 371, 20150275. 10.1098/rstb.2015.0275 PMC4843696 27114577

[ece39020-bib-0017] Eisenhauer, N. , Lanoue, A. , Strecker, T. , Scheu, S. , Steinauer, K. , Thakur, M. P. , & Mommer, L. (2017). Root biomass and exudates link plant diversity with soil bacterial and fungal biomass. Scientific Reports, 7(1), 44641. 10.1038/srep44641 28374800 PMC5379681

[ece39020-bib-0018] Fanin, N. , Kardol, P. , Farrell, M. , Nilsson, M.‐C. , Gundale, M. J. , & Wardle, D. A. (2019). The ratio of gram‐positive to gram‐negative bacterial PLFA markers as an indicator of carbon availability in organic soils. Soil Biology and Biochemistry, 128, 111–114. 10.1016/j.soilbio.2018.10.010

[ece39020-bib-0019] Fierer, N. , Schimel, J. P. , & Holden, P. (2003). Variations in microbial community composition through two soil depth profiles. Soil Biology and Biochemistry, 35(1), 167–176. 10.1016/S0038-0717(02)00251-1

[ece39020-bib-0020] Formaglio, G. , Veldkamp, E. , Damris, M. , Tjoa, A. , & Corre, M. D. (2021). Mulching with pruned fronds promotes the internal soil N cycling and soil fertility in a large‐scale oil palm plantation. Biogeochemistry, 154(1), 63–80. 10.1007/s10533-021-00798-4

[ece39020-bib-0021] Fox, J. , & Weisberg, S. (2019). An {R} companion to applied regression (3rd ed.). Sage.

[ece39020-bib-0022] Frostegard, A. , & Baath, E. (1996). The use of phospholipid fatty acid analysis to estimate bacterial and fungal biomass in soil. Biology and Fertility of Soils, 22(1–2), 59–65. 10.1007/BF00384433

[ece39020-bib-0023] Frostegård, A. , Tunlid, A. , & Bååth, E. (1993). Phospholipid fatty acid composition, biomass, and activity of microbial communities from two soil types experimentally exposed to different heavy metals. Applied and Environmental Microbiology, 59(11), 3605–3617. 16349080 10.1128/aem.59.11.3605-3617.1993 PMC182506

[ece39020-bib-0024] Frostegård, Å. , Tunlid, A. , & Bååth, E. (2011). Use and misuse of PLFA measurements in soils. Soil Biology and Biochemistry, 43, 1621–1625. 10.1016/j.soilbio.2010.11.021

[ece39020-bib-0025] Fujii, S. , Berg, M. P. , & Cornelissen, J. H. C. (2020). Living litter: Dynamic trait spectra predict Fauna composition. Trends in Ecology & Evolution, 35(10), 886–896. 10.1016/j.tree.2020.05.007 32522377

[ece39020-bib-0026] Gomez, E. J. , Delgado, J. A. , & Gonzalez, J. M. (2020). Environmental factors affect the response of microbial extracellular enzyme activity in soils when determined as a function of water availability and temperature. Ecology and Evolution, 10(18), 10105–10115. 10.1002/ece3.6672 33005367 PMC7520203

[ece39020-bib-0027] Grass, I. , Kubitza, C. , Krishna, V. V. , Corre, M. D. , Mußhoff, O. , Pütz, P. , Drescher, J. , Rembold, K. , Ariyanti, E. S. , Barnes, A. D. , Brinkmann, N. , Brose, U. , Brümmer, B. , Buchori, D. , Daniel, R. , Darras, K. F. A. , Faust, H. , Fehrmann, L. , Hein, J. , … Wollni, M. (2020). Trade‐offs between multifunctionality and profit in tropical smallholder landscapes. Nature Communications, 11(1), 1186. 10.1038/s41467-020-15013-5 PMC7055322 32132531

[ece39020-bib-0028] Grimbacher, P. S. , Edwards, W. , Liddell, M. J. , Nelson, P. N. , Nichols, C. , Wardhaugh, C. W. , & Stork, N. E. (2018). Temporal variation in abundance of leaf litter beetles and ants in an Australian lowland tropical rainforest is driven by climate and litter fall. Biodiversity and Conservation, 27(10), 2625–2640. 10.1007/s10531-018-1558-2

[ece39020-bib-0029] Grimbacher, P. S. , & Stork, N. E. (2009). Seasonality of a diverse beetle assemblage inhabiting lowland tropical rain Forest in Australia. Biotropica, 41(3), 328–337. 10.1111/j.1744-7429.2008.00477.x

[ece39020-bib-0030] Haruna, S. I. , Nkongolo, N. V. , Anderson, S. H. , Eivazi, F. , & Zaibon, S. (2018). In situ infiltration as influenced by cover crop and tillage management. Journal of Soil and Water Conservation, 73(2), 164–172. 10.2489/jswc.73.2.164

[ece39020-bib-0031] Hutchison, C. , Gravel, D. , Guichard, F. , & Potvin, C. (2018). Effect of diversity on growth, mortality, and loss of resilience to extreme climate events in a tropical planted forest experiment. Scientific Reports, 8(1), 15443. 10.1038/s41598-018-33670-x 30337582 PMC6193960

[ece39020-bib-0032] IPBES (2019). Global assessment report on biodiversity and ecosystem service. In Debating Nature's Value. IPBES.

[ece39020-bib-0033] Jacquemin, J. , Roisin, Y. , & Leponce, M. (2016). Spatio‐temporal variation in ant (Hymenoptera: Formicidae) communities in leaf‐litter and soil layers in a premontane tropical forest. Myrmecological News, 22, 129–139.

[ece39020-bib-0034] Joergensen, R. G. , & Scheu, S. (1999). Response of soil microorganisms to the addition of carbon, nitrogen and phosphorus in a forest rendzina. Soil Biology and Biochemistry, 31(6), 859–866. 10.1016/S0038-0717(98)00185-0

[ece39020-bib-0035] Johannes, R. C. , & Erland, B. (2009). Contrasting soil pH effects on fungal and bacterial growth suggest functional redundancy in carbon mineralization. Applied and Environmental Microbiology, 75(6), 1589–1596. 10.1128/AEM.02775-08 19151179 PMC2655475

[ece39020-bib-0036] Kempson, D. , Lloyd, M. , & Ghelardi, R. (1963). A new extractor for woodland litter. Pedobiologia, 3(1), 1–21.

[ece39020-bib-0037] Keupp, L. , Pollinger, F. , & Paeth, H. (2017). Assessment of future ENSO changes in a CMIP3/CMIP5 multi‐model and multi‐index framework. International Journal of Climatology, 37(8), 3439–3451. 10.1002/joc.4928

[ece39020-bib-0038] Ko, D. , Yoo, G. , Yun, S.‐T. , Jun, S.‐C. , & Chung, H. (2017). Bacterial and fungal community composition across the soil depth profiles in a fallow field. Journal of Ecology and Environment, 41(1), 34. 10.1186/s41610-017-0053-0

[ece39020-bib-0039] Kramer, C. , & Gleixner, G. (2008). Soil organic matter in soil depth profiles: Distinct carbon preferences of microbial groups during carbon transformation. Soil Biology and Biochemistry, 40(2), 425–433. 10.1016/j.soilbio.2007.09.016

[ece39020-bib-0040] Krashevska, V. , Klarner, B. , Widyastuti, R. , Maraun, M. , & Scheu, S. (2015). Impact of tropical lowland rainforest conversion into rubber and oil palm plantations on soil microbial communities. Biology and Fertility of Soils, 51(6), 697–705. 10.1007/s00374-015-1021-4

[ece39020-bib-0041] Krashevska, V. , Sandmann, D. , Maraun, M. , & Scheu, S. (2012). Consequences of exclusion of precipitation on microorganisms and microbial consumers in montane tropical rainforests. Oecologia, 170(4), 1067–1076. 10.1007/s00442-012-2360-6 22614263 PMC3496542

[ece39020-bib-0042] Kunert, N. , & Cárdenas, A. M. (2015). Are mixed tropical tree plantations more resistant to drought than monocultures? Forests, 6(6), 2029–2046. 10.3390/f6062029

[ece39020-bib-0043] Kurzatkowski, D. , Martius, C. , Höfer, H. , Garcia, M. , Förster, B. , Beck, L. , & Vlek, P. (2004). Litter decomposition, microbial biomass and activity of soil organisms in three agroforestry sites in Central Amazonia. Nutrient Cycling in Agroecosystems, 69(3), 257–267. 10.1023/B:FRES.0000035196.19804.13

[ece39020-bib-0044] Lan, G. , Yang, C. , & Wu, Z. (2021). Network complexity of rubber plantations is lower than tropical forests for soil bacteria but not fungi. SOIL Discuss, 2021, 1–41. 10.5194/soil-2021-98

[ece39020-bib-0045] Lepcha, N. T. , & Devi, N. B. (2020). Effect of land use, season, and soil depth on soil microbial biomass carbon of eastern Himalayas. Ecological Processes, 9(1), 65. 10.1186/s13717-020-00269-y

[ece39020-bib-0046] Liu, W. , Zhang, Z. , & Wan, S. (2009). Predominant role of water in regulating soil and microbial respiration and their responses to climate change in a semiarid grassland. Global Change Biology, 15(1), 184–195. 10.1111/j.1365-2486.2008.01728.x

[ece39020-bib-0047] Malik, A. A. , Chowdhury, S. , Schlager, V. , Oliver, A. , Puissant, J. , Vazquez, P. G. M. , Jehmlich, N. , von Bergen, M. , Griffiths, R. I. , & Gleixner, G. (2016). Soil fungal:Bacterial ratios are linked to altered carbon cycling. Frontiers in Microbiology, 7, 1247. https://www.frontiersin.org/article/10.3389/fmicb.2016.01247 27555839 10.3389/fmicb.2016.01247 PMC4977315

[ece39020-bib-0048] Marín, L. , Philpott, S. M. , De la Mora, A. , Ibarra Núñez, G. , Tryban, S. , & Perfecto, I. (2016). Response of ground spiders to local and landscape factors in a Mexican coffee landscape. Agriculture, Ecosystems & Environment, 222, 80–92. 10.1016/j.agee.2016.01.051

[ece39020-bib-0049] Martius, C. , Höfer, H. , Garcia, M. V. B. , Römbke, J. , Förster, B. , & Hanagarth, W. (2004). Microclimate in agroforestry systems in Central Amazonia: Does canopy closure matter to soil organisms? Agroforestry Systems, 60(3), 291–304. 10.1023/B:AGFO.0000024419.20709.6c

[ece39020-bib-0050] Meijide, A. , Badu, C. S. , Moyano, F. , Tiralla, N. , Gunawan, D. , & Knohl, A. (2018). Impact of forest conversion to oil palm and rubber plantations on microclimate and the role of the 2015 ENSO event. Agricultural and Forest Meteorology, 252, 208–219. 10.1016/j.agrformet.2018.01.013

[ece39020-bib-0051] Miettinen, J. , Hooijer, A. , Shi, C. , Tollenaar, D. , Vernimmen, R. , Liew, S. C. , Malins, C. , & Page, S. E. (2012). Extent of industrial plantations on southeast Asian peatlands in 2010 with analysis of historical expansion and future projections. GCB Bioenergy, 4(6), 908–918. 10.1111/j.1757-1707.2012.01172.x

[ece39020-bib-0052] Millard, S. P. (2013). EnvStats: An R package for environmental statistics. Springer. https://www.springer.com

[ece39020-bib-0053] Montine, P. S. M. , Viana, N. F. , Almeida, F. S. , Dátillo, W. F. D. C. , Santanna, A. S. , Martins, L. , & Vargas, A. B. (2014). Seasonality of Epigaeic ant communities in a Brazilian Atlantic rainforest. Sociobiology, 61(2), 178–183. 10.13102/sociobiology.v61i2.178-183

[ece39020-bib-0054] Morriën, E. , Hannula, S. E. , Snoek, L. B. , Helmsing, N. R. , Zweers, H. , de Hollander, M. , Soto, R. L. , Bouffaud, M.‐L. , Buée, M. , Dimmers, W. , Duyts, H. , Geisen, S. , Girlanda, M. , Griffiths, R. I. , Jørgensen, H.‐B. , Jensen, J. , Plassart, P. , Redecker, D. , Schmelz, R. M. , … van der Putten, W. H. (2017). Soil networks become more connected and take up more carbon as nature restoration progresses. Nature Communications, 8(1), 14,349. 10.1038/ncomms14349 PMC5309817 28176768

[ece39020-bib-0055] Nepstad, D. C. , Tohver, I. M. , Ray, D. , Moutinho, P. , & Cardinot, G. (2007). Mortality of large trees and lianas following experimental drought in an Amazon forest. Ecology, 88(9), 2259–2269. 10.1890/06-1046.1 17918404

[ece39020-bib-0056] Oksanen, J. , Legendre, P. , O'Hara, B. , Stevens, M. H. H. , Oksanen, M. J. , & Suggests, M. (2020). Vegan: Community Ecology Package . R package version 2.5–7. https://cran.r‐project.org/package=vegan

[ece39020-bib-0057] Paterson, R. R. M. , Kumar, L. , Shabani, F. , & Lima, N. (2017). World climate suitability projections to 2050 and 2100 for growing oil palm. The Journal of Agricultural Science, 155(5), 689–702. 10.1017/S0021859616000605

[ece39020-bib-0058] Phillips, O. L. , van der Heijden, G. , Lewis, S. L. , López‐González, G. , Aragão, L. E. O. C. , Lloyd, J. , Malhi, Y. , Monteagudo, A. , Almeida, S. , Dávila, E. A. , Amaral, I. , Andelman, S. , Andrade, A. , Arroyo, L. , Aymard, G. , Baker, T. R. , Blanc, L. , Bonal, D. , de Oliveira, Á. C. A. , … Vilanova, E. (2010). Drought–mortality relationships for tropical forests. New Phytologist, 187(3), 631–646. 10.1111/j.1469-8137.2010.03359.x 20659252

[ece39020-bib-0059] Pinheiro, J. , Bates, D. , DebRoy, S. , Sarkar, D. R. , & R Core Team . (2020). nlme: Linear and Nonlinear Mixed Effects Models . R package version 3.1–148. https://CRAN.R‐project.org/package=nlme.

[ece39020-bib-0060] Pirker, J. , Mosnier, A. , Kraxner, F. , Havlík, P. , & Obersteiner, M. (2016). What are the limits to oil palm expansion? Global Environmental Change, 40, 73–81. 10.1016/j.gloenvcha.2016.06.007

[ece39020-bib-0061] Pollierer, M. M. , Langel, R. , Körner, C. , Maraun, M. , & Scheu, S. (2007). The underestimated importance of belowground carbon input for forest soil animal food webs. Ecology Letters, 10(8), 729–736. 10.1111/j.1461-0248.2007.01064.x 17594428

[ece39020-bib-0062] Potapov, A. M. , Klarner, B. , Sandmann, D. , Widyastuti, R. , & Scheu, S. (2019). Linking size spectrum, energy flux and trophic multifunctionality in soil food webs of tropical land‐use systems. Journal of Animal Ecology, 1365–2656, 13027. 10.1111/1365-2656.13027 31111468

[ece39020-bib-0063] Rembold, K. , Mangopo, H. , Tjitrosoedirdjo, S. S. , & Kreft, H. (2017). Plant diversity, forest dependency, and alien plant invasions in tropical agricultural landscapes. Biological Conservation, 213(March), 234–242. 10.1016/j.biocon.2017.07.020

[ece39020-bib-0064] Rizqulloh, M. N. , Drescher, J. , Hartke, T. R. , Potapov, A. , Scheu, S. , Hidayat, P. , & Widyastuti, R. (2021). Effects of rainforest transformation to monoculture cash crops on soil living ants (Formicidae) in Jambi Province, Sumatra, Indonesia. IOP Conference Series: Earth and Environmental Science, 771(1), 12031. 10.1088/1755-1315/771/1/012031

[ece39020-bib-0065] Ruess, L. , & Chamberlain, P. M. (2010). The fat that matters: Soil food web analysis using fatty acids and their carbon stable isotope signature. Soil Biology and Biochemistry, 42(11), 1898–1910. 10.1016/j.soilbio.2010.07.020

[ece39020-bib-0066] Sabajo, C. R. , le Maire, G. , June, T. , Meijide, A. , Roupsard, O. , & Knohl, A. (2017). Expansion of oil palm and other cash crops causes an increase of the land surface temperature in the Jambi province in Indonesia. Biogeosciences, 14(20), 4619–4635. 10.5194/bg-14-4619-2017

[ece39020-bib-0067] Scheu, S. (1992). Automated measurement of the respiratory response of soil microcompartments: Active microbial biomass in earthworm faeces. Soil Biology and Biochemistry, 24, 1113–1118.

[ece39020-bib-0068] Seuradge, B. J. , Oelbermann, M. , & Neufeld, J. D. (2017). Depth‐dependent influence of different land‐use systems on bacterial biogeography. FEMS Microbiology Ecology, 93(2), fiw239. 10.1093/femsec/fiw239 27915285

[ece39020-bib-0069] Sumarga, E. , & Hein, L. (2016). Benefits and costs of oil palm expansion in Central Kalimantan, Indonesia, under different policy scenarios. Regional Environmental Change, 16(4), 1011–1021. 10.1007/s10113-015-0815-0 27429582 PMC4927089

[ece39020-bib-0070] Susanti, W. I. , Bartels, T. , Krashevska, V. , Widyastuti, R. , Deharveng, L. , Scheu, S. , & Potapov, A. (2021). Conversion of rainforest into oil palm and rubber plantations affects the functional composition of litter and soil collembola. Ecology and Evolution, 11(15), 10686–10708. 10.1002/ece3.7881 34367606 PMC8328430

[ece39020-bib-0071] Tao, H.‐H. , Snaddon, J. L. , Slade, E. M. , Henneron, L. , Caliman, J.‐P. , & Willis, K. J. (2018). Application of oil palm empty fruit bunch effects on soil biota and functions: A case study in Sumatra, Indonesia. Agriculture, Ecosystems & Environment, 256, 105–113. 10.1016/j.agee.2017.12.012

[ece39020-bib-0072] Taylor, A. R. , Schröter, D. , Pflug, A. , & Wolters, V. (2004). Response of different decomposer communities to the manipulation of moisture availability: Potential effects of changing precipitation patterns. Global Change Biology, 10(8), 1313–1324. 10.1111/j.1365-2486.2004.00801.x

[ece39020-bib-0073] Wagg, C. , Bender, S. F. , Widmer, F. , & van der Heijden, M. G. (2014). Soil biodiversity and soil community composition determine ecosystem multifunctionality. Proceedings of the National Academy of Sciences of the United States of America, 111(14), 5266–5270. 10.1073/pnas.1320054111 24639507 PMC3986181

[ece39020-bib-0074] Warton, D. I. , & Hui, F. K. C. (2011). The arcsine is asinine: The analysis of proportions in ecology. Ecology, 92(1), 3–10. 10.1890/10-0340.1 21560670

[ece39020-bib-0075] Yin, R. , Gruss, I. , Eisenhauer, N. , Kardol, P. , Thakur, M. P. , Schmidt, A. , Xu, Z. , Siebert, J. , Zhang, C. , Wu, G.‐L. , & Schädler, M. (2019). Land use modulates the effects of climate change on density but not community composition of collembola. Soil Biology and Biochemistry, 138(107), 598. 10.1016/j.soilbio.2019.107598

[ece39020-bib-0076] Yin, R. , Siebert, J. , Eisenhauer, N. , & Schädler, M. (2020). Climate change and intensive land use reduce soil animal biomass via dissimilar pathways. ELife, 9, e54749. 10.7554/eLife.54749 32718434 PMC7386910

[ece39020-bib-0077] Yu, S. , Mo, Q. , Li, Y. , Li, Y. , Zou, B. , Xia, H. , Li, Z. , & Wang, F. (2019). Changes in seasonal precipitation distribution but not annual amount affect litter decomposition in a secondary tropical forest. Ecology and Evolution, 9(19), 11344–11352. 10.1002/ece3.5635 31641477 PMC6802026

[ece39020-bib-0078] Zelles, L. (1997). Phospholipid fatty acid profiles in selected members of soil microbial communities. Chemosphere, 35, 275–294. 9232001 10.1016/s0045-6535(97)00155-0

[ece39020-bib-0079] Zelles, L. (1999). Fatty acid patterns of phospholipids and lipopolysaccharides in the characterisation of microbial communities in soil: a review. Biology and Fertility of Soils, 29(2), 111–129. 10.1007/s003740050533

[ece39020-bib-0080] Zuraidah, Y. , Aminuddin, H. , Jamal, T. , Jamarei, O. , Osumanu, H. A. , & Mohamadu, B. J. (2010). Oil palm (Elaeis guineensis) roots response to mechanization in Bernam series soil. American Journal of Applied Sciences, 7(3), 343–348. 10.3844/ajassp.2010.343.348

[ece39020-bib-0081] Zuur, A. , Ieno, E. N. , Walker, N. , Saveliev, A. A. , & Smith, G. M. (2009). Mixed effects models and extensions in ecology with R. Springer Science & Business Media.

