# Supplementary material for: Land‐use change shifts and magnifies seasonal variations of the decomposer system in lowland tropical landscapes
Source: Ecol Evol. 2022 Jun 17;12(6):e9020. doi: 10.1002/ece3.9020 (PMC9205671; doi:10.1002/ece3.9020)
Supplement: Supplementary file 1 — Figures S1‐S2 [file ECE3-12-e9020-s001.docx]

**Supplementary Figure 1 and 2.**


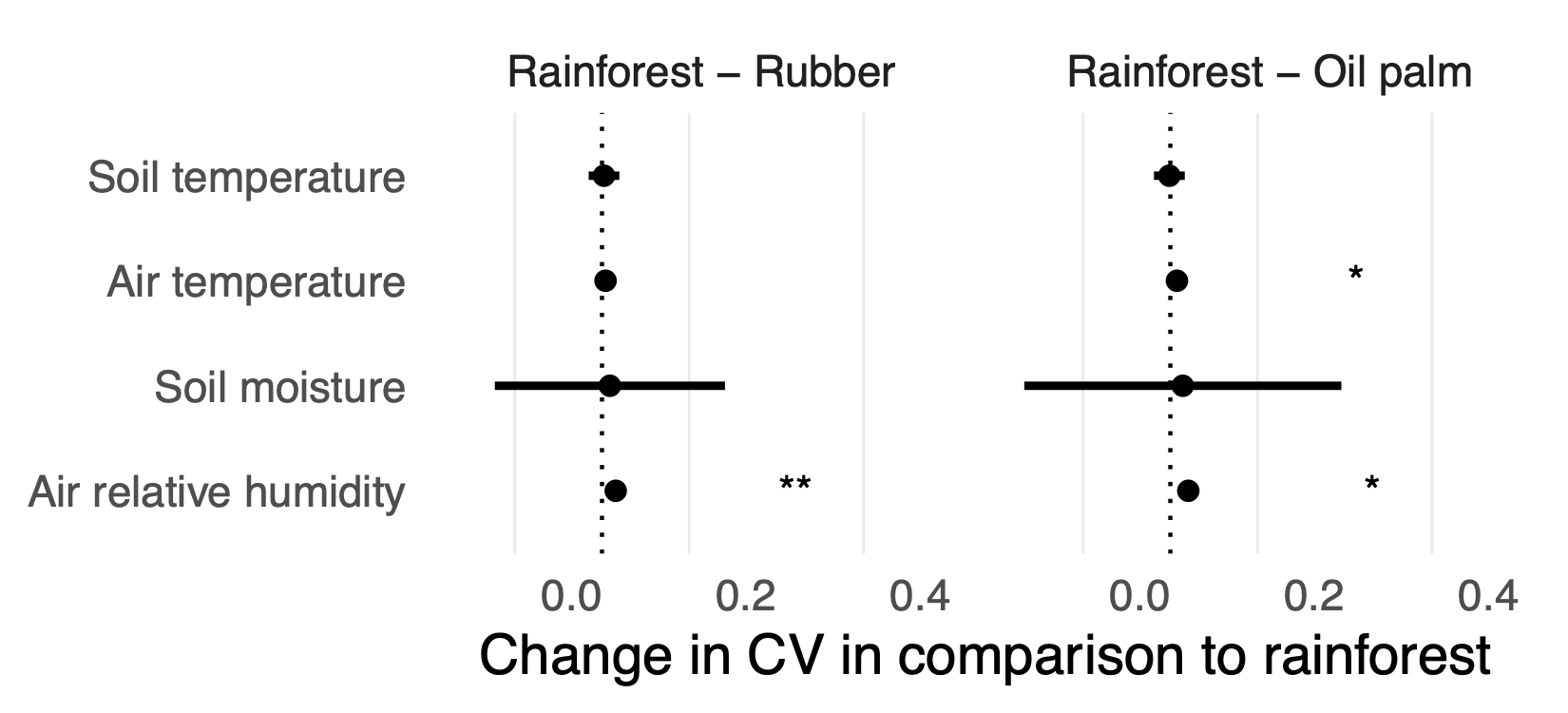


**Fig. Supp 1.** Difference in the magnitude of seasonal variations in below-canopy air relative humidity, air temperature and in soil moisture and temperature (30 cm depth) between rainforest and plantations. Changes in the coefficients of variation (CV) are shown for rubber (left) and oil palm (right). Confidence intervals that do not overlap with zero indicate significantly higher CV in the plantation than in the rainforest (shift to the right).


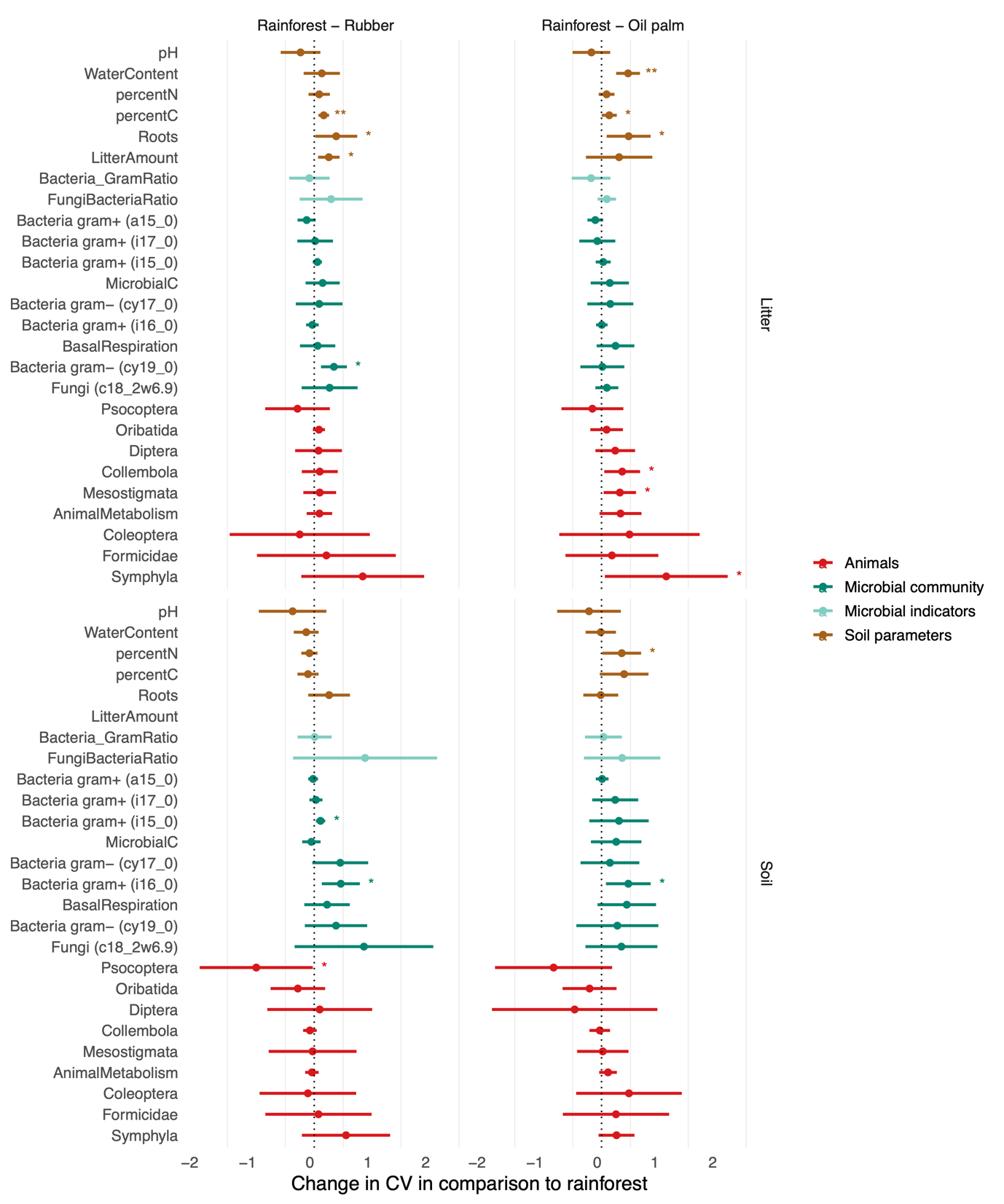


**Fig. Supp 2.** Difference in the magnitude of seasonal variation of animal groups, microbial community and soil parameters between rainforest and plantations. Changes in the coefficients of variation (CV) are shown for rubber (left) and oil palm (right). Confidence intervals that do not overlap with zero indicate significantly higher CV in the plantation than in the rainforest (shift to the right).
